# RUNX regulated immune-associated genes predicts prognosis in breast cancer

**DOI:** 10.3389/fgene.2022.960489

**Published:** 2022-08-26

**Authors:** Jingyue Fu, Handong Sun, Feng Xu, Rui Chen, Xinyang Wang, Qiang Ding, Tiansong Xia

**Affiliations:** Jiangsu Breast Disease Center, The First Affiliated Hospital with Nanjing Medical University, Nanjing, China

**Keywords:** runx, immune, TCGA, prognostic model, breast cancer

## Abstract

**Background:** Breast cancer is the most common malignant tumor in women. RUNX family has been involved in the regulation of different carcinogenic processes and signaling pathways with cancer, which is closely related to immunity and prognosis of various tumors, and also plays an important role in the development and prognosis of breast cancer.

**Methods:** We discovered the expression of RUNX family through GEPIA Dataset and then evaluated the relationship between RUNX family and immune-related genes and the prognosis of breast cancer through analyzing TCGA database. A prognostic model was established and verified *via* cox proportional hazards regression model using R packages. We evaluated the accuracy of the prognostic model by Kaplan-Meier curves and receiver operating characteristic (ROC) curves. Additionally, we obtained the relationship between the RUNX family and immune infiltration by TIMER database. Finally, the dual luciferase reporter assay was used to verify the regulation of RUNX3 on potential target genes ULBP2 and TRDV1, and the effects of ULBP2 and TRDV1 on the growth of breast cancer cells were explored by CCK-8, colony formation and wound healing assays.

**Results:** We screened out RUNX family-regulated immune-related genes associated with the prognosis of breast cancer. These predictors included PSME2, ULBP2, IL-18, TSLP, NPR3, TRDV1. Then a prognosis-related risk score model was built using the independent risk factors to provide a clinically appropriate method predicting the overall survival (OS) probability of the patients with breast cancer. In addition, a further research was made on the functions of high risk immune gene ULBP2 and low risk immune gene TRDV1 which regulated by RUNX3, the results showed that down-regulation of ULBP2 suppressed breast cancer cell proliferation and TRDV1 had the opposite functions. The prognostic model we constructed could promote the development of prognostic, and was associated with lower immune infiltration.

**Conclusion:** The expression of RUNX family was closely related to the prognosis of breast cancer. At the same time, RUNX family could modulate the functions of immune-related genes, and affect the development and prognosis of breast cancer. These immune-related genes regulated by RUNX family could be promising prognostic biomarkers and therapeutic targets in breast cancer.

## Introduction

Breast cancer is the most common malignant tumor threatening women’s health. The latest data from the World Health Organization reveals that breast cancer is the most commonly diagnosed cancer in females (11.7% of total cases), no matter in the developed city or developing countries (Sung et al., 2021). Despite the huge advancements in its early diagnosis and treatment, the prognosis of some breast cancer patients is still poor resulting of the complex nature of breast cancer. At present, although plenty of biomarkers and algorithms have been employed to predict the prognosis of patients with breast cancer, it inevitably leads to the deviation of relevant prediction methods due to the particularity of the pathogenesis, development, and metastasis of breast cancer. Therefore, it is imperative to search for highly sensitive and specific prognostic markers to explore new targets and improve the prognosis of breast cancer patients (Sun et al., 2019). At present, many gene family members have been identified as being involved in the tumor progression of various cancers. For example, members of the Arp2/3 family play an important role in predicting the prognosis of liver cancer and are associated with immune cell infiltration (Huang et al., 2021). The role of E2F transcription factors in the tumorigenesis and prognosis of several cancers has also been partially confirmed (Sun et al., 2019).

The runt-related transcription factors (RUNX) belong to a transcription factors family (Mevel et al., 2019). To date, three RUNX transcription factors (RUNX1, RUNX2, RUNX3) have been identified as master regulators of important embryonic developmental programs in mammalian cells. They could regulate cell proliferation, and differentiation in different cancers (Otalora-Otalora et al., 2019). RUNXs have been identified as being involved in the progression of various tumors. Many studies have shown that abnormal expression of RUNXs was associated with the proliferation and invasion of cancers and the role of RUNX factors has been found to be two-sided in cancer biology (Liu et al., 2018; Ashe et al., 2021). They have been found to exert either oncogenic or tumor suppressive roles in the development of hematopoietic cancer as well as solid tumors (Ito et al., 2015). In recent years, a growing number of studies have suggested a role for RUNX genes in breast cancer. RUNXs were supposed to have complex and distinct roles in human breast cancer, especially in tumor invasion and metastasis (Rooney et al., 2017; Fritz et al., 2020). RUNX1 has been identified as a critical regulator of definitive hematopoiesis in early studies (Sood et al., 2017). But Andrew J. Fritz et al. (Fritz et al., 2020) discovered that RUNXs have oncogenic potential or tumor suppressor abilities during the oncogenic process, suggesting their importance as biomarkers of breast cancer. It was reported that RUNX2 is a critical regulator that can maintain osteoblast development and osteoclast process (Komori, 2020; Qin et al., 2020). Recent studies by Selvamurugan *et al* (Vishal et al., 2017) established that RUNX2 is indirectly involved in the metastasis of breast cancer by regulating the genes related to metastasis and invasion. RUNX3, another member of RUNX family, which is located on human chromosome 1p36 is involved in the development of gastric cancer and hepatocellular carcinoma (Ni et al., 2020; Song et al., 2020). Some studies founded that RUNX3 is inactivated in several tumors including breast cancer and it is identified as a tumor suppressor to reduce the initiation and progression of breast cancer (Huang et al., 2012; Liu et al., 2020).

The functional role of RUNX was often closely related to influencing immune infiltration, and some studies founded the functions of RUNX proteins mainly on lymphoid lineage cells (Seo and Taniuchi, 2020). But the importance of the RUNX factors to immunity has been obscured for historic, technical, and conceptual reasons, and has rarely been reported in recent years (Voon et al., 2015). In the past years, studies founded that the RUNX factors are involved in the development of T cells in the thymus, hematopoietic cell development, and chromatin remodeling (Bowcock, 2005). For instance, RUNX1 and RUNX3 are further involved in the maturation of naive CD4^+^ T cells into various effector T-cell lineages. RUNX3 expression increases with a corresponding decrease in RUNX1 expression when T helper type 1 (Th1) differentiation (Ito et al., 2015). The RUNX factors could also regulate adhesion complexes to promote the migration of macrophages and monocytes to the sites of infection to perform their phagocytic functions (Puig-Kröger et al., 2003; Estecha et al., 2012). In summary, the RUNX factors are involved not only in tumor growth and spread, but also in the development, organization, and function of the mammalian immune system (Seo and Taniuchi, 2020).

However, the distinct functions of the RUNX factors and the immune-related genes is regulated in breast cancer have not been fully elucidated. And to our knowledge, the prognostic analyses of breast cancer, which are based on the expression of immune-related genes regulated by the RUNX family have not been completely studied or reported. In recent research, some researchers have found that RUNX family plays an important role in the regulation of immune cell development and function (Gao and Zhou, 2021). In this study, we further explored the respective roles of the RUNX family members in breast cancer and found that it was related to the immune invasion. Meanwhile, we established a prognostic model based on immune-related genes regulated by RUNX family to predict breast cancer survival, and may provide potential biomarkers or therapeutic targets for the diagnosis and treatment of early breast cancer.

## Methods

### GEPIA dataset

The RNA sequencing expression data from The Cancer Genome Atlas and the Genotype Tissue Expression (GTEx) projects were analyzed with GEPIA (http://gepia.cancer-pku.cn/index.html), a newly developed interactive web tool, to discover the RUNX family expression in breast cancer by a standard processing pipeline (Tang et al., 2017). The survival analysis was also performed *via* a Kaplan-Meier curve for further verification by GEPIA. In addition, RUNX family protein levels were analyzed by the Human Protein Atlas database (HPA) (https://www.proteinatlas.org/) to verify whether the expression of the mRNA and protein levels matched (Uhlén et al., 2015).

### The Kaplan-Meier plotter

The prognostic value of the RUNX family mRNA expression was analyzed through an online database, Kaplan-Meier Plotter (http://kmplot.com/analysis/), which provided gene expression data and survival information of 1,809 breast cancer patients. And the clinical relationships between gene expression of the RUNX family and survival information including recurrence-free survival (RFS) and overall survival (OS) were assessed by Kaplan-Meier Plotter. The prognostic value of the RUNX family, such as 95% confidence intervals (CIs), hazard ratio (HR), and *p* value could be automatically calculated based on the RNA expression (high vs low) of RUNX family genes.

### The cancer genome atlas data and cBioPortal

The RUNX family mRNA expression data of breast cancer were downloaded from The Cancer Genome Atlas database (https://genome-cancer.ucsc.edu/). R package limma was used to normalize and differential expression analysis of these data from the TCGA database. P value < 0.05 was considered statistically significant. Additionally, cBioPortal (http://www.cbioportal.org), a comprehensive web resource, was used for further analyses of the RUNX family. According to The Cancer Genome Atlas (TCGA) database, co-expression and network module of the RUNX family were calculated from cBioPortal.

### String and tumor immunity analyses

STRING (https://string-db.org/), a website about protein interaction, was performed to achieve a comprehensive and objective global network and then present them with a unique set of computational predictions. The PPI network analysis was performed to collect and integrate the different expressions of the RUNX family and potential interactions using STRING. *p* < 0.05 was considered statistically significant. The abundances of B, CD4^+^ T, CD8^+^ T, NK, and dendritic cells and macrophages were estimated through the TIMER (https://cistrome.shinyapps.io/timer/), which is a friendly web interface for the user. In addition, the correlation of the RUNX family expression with immune cell infiltration scores was revealed by TIMER. The RUNX family was conducted to input using the Gene module and generated scatterplots to visualize the correlation of their expression with immune infiltration level in breast cancer.

### Prognostic model construction

Using univariate and multivariate Cox regression, six independent genes associated with RUNX were identified. Next, the risk score model was structured based on the expression levels and coefficients of the six hub Coxs. The risk score of each breast cancer patient was counted using the next formula: Risk score = β1*Exp1 + β2*Exp2 + βi*Expi, where *β* manifests the coefficient value of the independent prognosis-associated RUNXs, Exp manifests the expression level of the independent prognosis-associated Cox.

### Validating the performance of the prognostic model

According to the median risk score, the breast cancer patients were divided into high-and low-risk groups. The difference in survival between the two groups was assessed by the Kaplan-Meier method using log-rank tests. Additionally, receiver operating characteristic (ROC) curves, were used for confirming the accuracy of the prognostic model.

### Tissue samples and cell lines

Fresh breast cancer tissues and adjacent normal tissues were collected from the First Affiliated Hospital with Nanjing Medical University. Experienced pathologist immediately isolated the primary tumor area and morphologically normal surgical margin tissue after resection from each patient and stored in liquid nitrogen until use. The study was approved by the institutional ethical committee of the First Affiliated Hospital with Nanjing Medical University. Informed written consents were obtained from patients recruited in this study. The human breast cancer cell line MDA-MB-231 was cultured in DMEM supplemented with 10% fetal bovine serum (Invitrogen, Canada) at 37°C under 5% CO ^2^ in a humidified incubator.

### Luciferase report assay

Luciferase reporter plasmids were constructed by iGeneBio (Guangzhou, China). Based on the chemiluminescence reaction between luciferase and substrate, mutant and wild-type ULBP2 and TRDV1 of the putative binding sites were cloned into a luciferase vector and co-transfected with RUNX3 mimics into breast cancer cells. After 48 h, cells were harvested for luciferase activity analysis using the Dual-Luciferase Reporter Assay System (Vazyme, China). The assay was repeated at least three times in independent experiments.

### Cell transfection

Transfections were carried out using the Lipofectamine 3000 Reagent (Invitrogen, Canada) following the manufacturer’s protocol. The small interfering RNAs (siRNAs) targeting ULBP2, TRDV1, and their negative controls were purchased from RiboBio (Guangzhou, China). MDA-MB-231 cells were plated in 96-well plates, when cells reached a confluence of 80%, they were transfected with the fragments or plasmids (0.2 µg/well) by Lipo3000 reagent, according to the protocol. After incubation at 37°C for 24 h, the transfected cells were harvested for subsequent experiments.

### CCK-8 assay

Cells were seeded into 96-well plates at a concentration of 2000 cells per well. According to the CCK8 (Beyotime, China) manufacturer’s instructions, cellular viability was determined by measuring the absorbance of the converted dye at 450 nm 2 h after adding CCK8. Measurements were taken every 24 h for 5 consecutive days.

### EdU assay

Cell-Light EdU Apollo *In Vitro* Kit (Ribobio, China) for EdU assay was utilized to compare the growth ability of the transfected breast cancer cell. MDA-MB-231 cell lines were transfected with ULBP2 and TRDV1 mimics control or inhibitor, then 5 × 10 ^3^ transfected cells were transferred into each well of the 96-well plate. EdU medium was added to each well to incubate the cells for two hours. We captured the images with a fluorescence microscope after the fixation, permeabilization, and staining.

### Colony formation assay

Cells transfected with ULBP2 and TRDV1 mimics control or inhibitor were plated at a density of 500 cells/6 cm dish. After 2 weeks of culture, colonies resulting from the surviving cells were fixed with 3.7% methanol, stained with 0.1% crystal violet, and counted.

### Wound healing assay

MDA-MB-231 cells were seeded in a six-well dish and incubated for 24 h; The monolayer was then scratched with pipette tips and washed with PBS. Photographs were taken at 0 and 48 h in an inverted microscope. The wound-healing rate was calculated as follows: Wound-healing rate = (Original wound area—area at detection)/original wound area × 100%.

## Results

### The transcription levels of RUNX family in breast cancer

**FIGURE 1:**
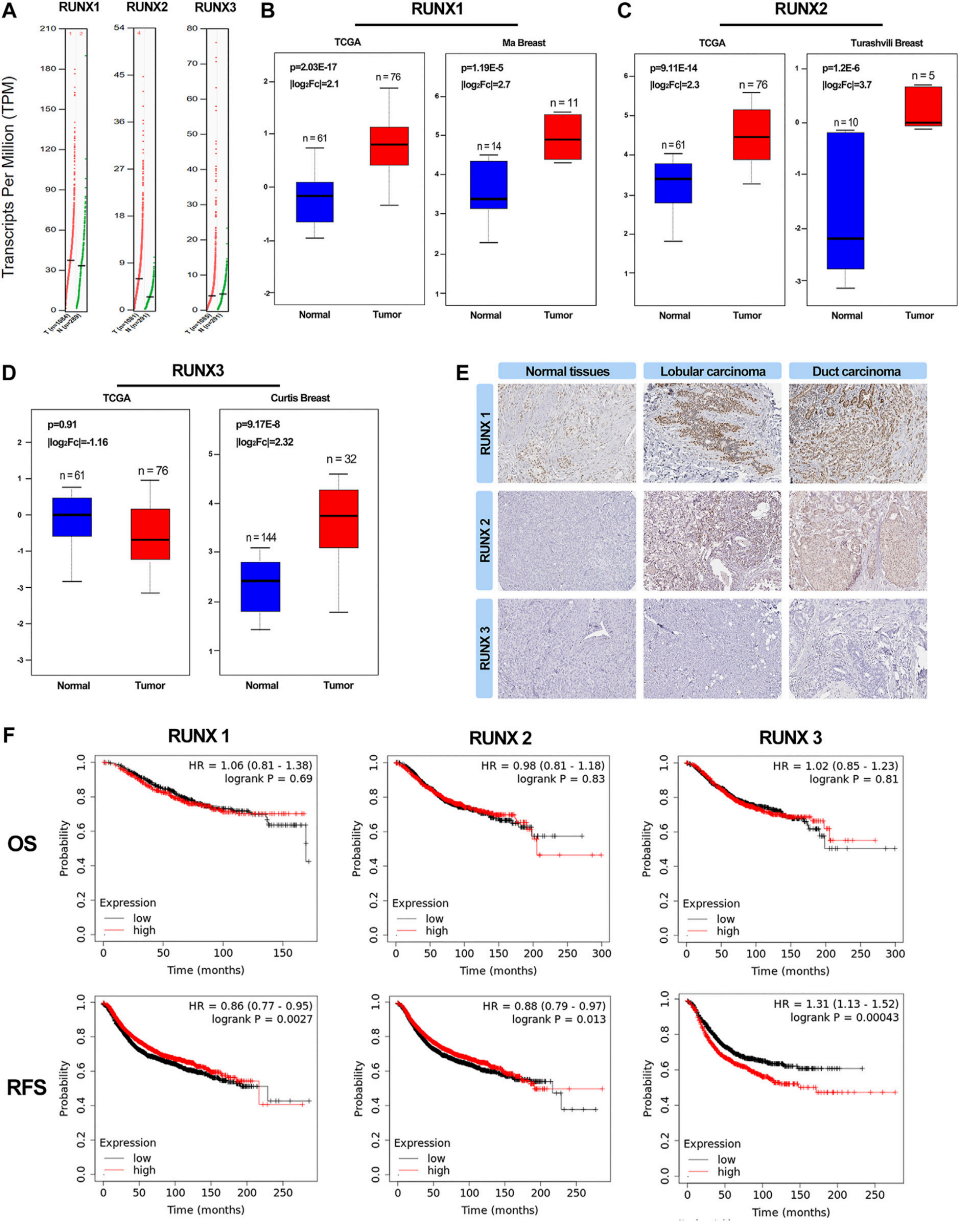
Expression of RUNXs in breast cancer and normal samples (GEPIA). Scatter diagram demonstrated that the expression levels of RUNX1, RUNX2 and RUNX3 were higher in breast cancer tissues than in normal tissues (*p* <0.05) **(A)**. A boxplot of the RUNXs expression profiles in breast cancer and normal samples using TCGA and other data **(B–D)**. Validation of the expression of RUNX proteins in breast cancer and normal tissues in the Human Protein Atlas (HPA) database **(E)**. Kaplan-Meier survival curve of OS and RFS for breast cancer in RUNXs. Kaplan-Meier analysis and log-rank test demonstrated a significant difference in RFS among the three groups (*p* <0.05) **(F)**.

To explore the distinct expressions of RUNX family in breast cancer patients, we analyzed the data in the public databases GEPIA. The results revealed that the expression levels of RUNX1, RUNX2, RUNX3 were higher in tumor tissues than in normal tissues, especially the expression of RUNX2 and RUNX3 (Figure 1A). The expression level of RUNX family in tumor tissues was higher than that in normal breast tissues while there was no difference in the expression of RUNX3 in the TCGA database (Figures 1B–D). Then, we further probed the protein expression of the RUNX family in the Human Protein Profiles, the results indicated that protein expression levels of RUNX family were higher in both ductal and lobular breast cancers than in normal (Figure 1E). In addition, the GEPIA tool was used to analyze the correlation between the expression of RUNX family and the overall survival (OS) and recurrence-free survival (RFS) of breast cancer. We founded that the expression of RUNX family was associated with RFS and could affect the prognosis of breast cancer (Figure 1F).

### Interaction analyses of RUNX family in breast cancer patients

**FIGURE 2:**
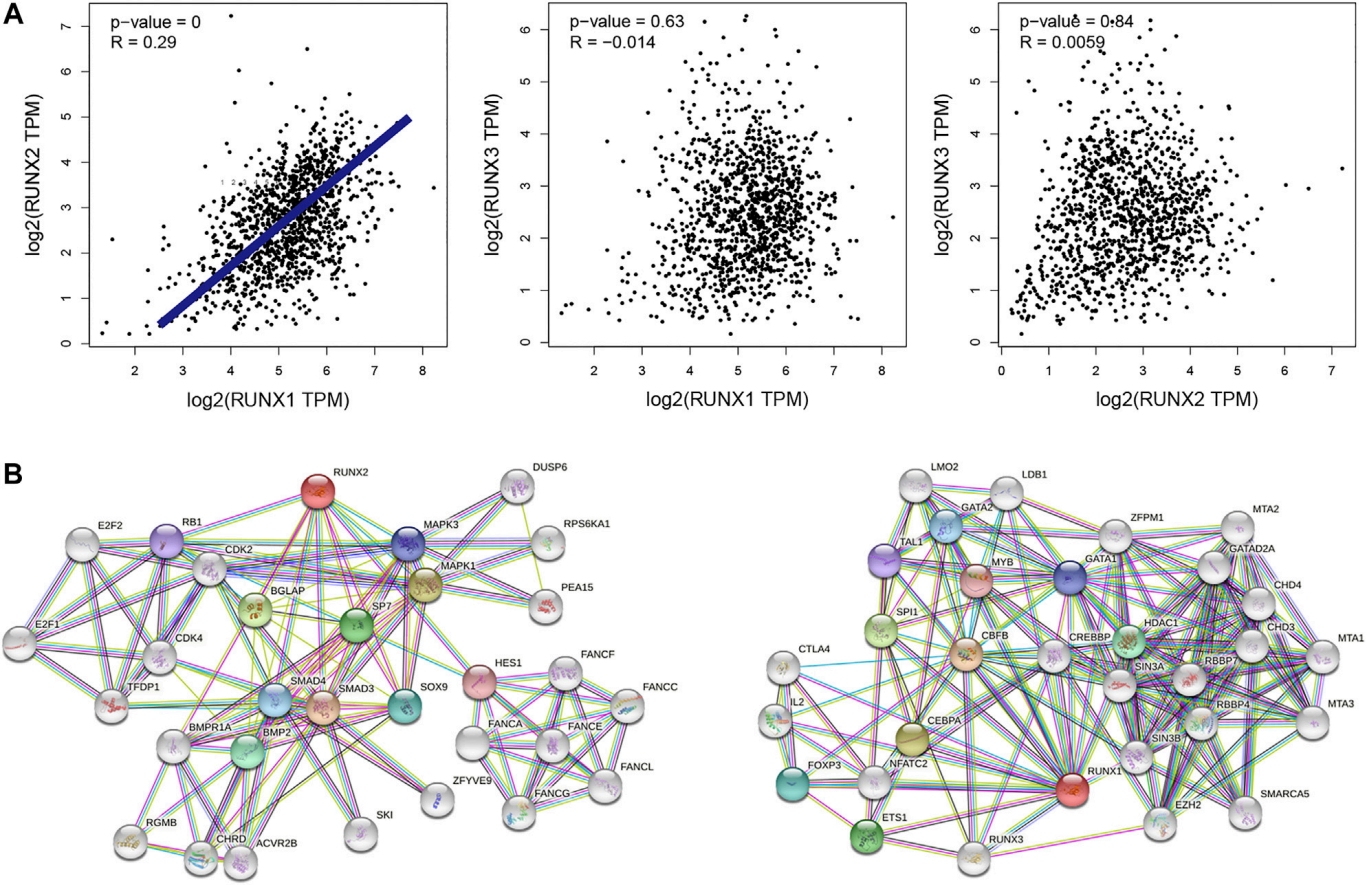
Interaction analyses of RUNX family in breast cancer **(A)**. The network for RUNX family in breast cancer and the part of most frequently altered neighbor genes **(B)**.

The correlations of RUNX family with each other were analyzed *via* the cBioPortal online tool for breast cancer according to their mRNA expressions. The results showed a noteworthy and positive relationship between RUNX1 and RUNX2 (Figure 2A). To explore the potential interactions among RUNX family, we conducted a protein-protein interaction PPI network analysis of the differentially expressed RUNX family with STRING. As the findings suggested, the neighbor genes such as MAPK1, MAPK3, BGLAP, ETS1, and CBFB had a close association with RUNX family alterations in the RUNX Family (Figure 2B).

### RUNX family regulated immune-related genes

**FIGURE 3:**
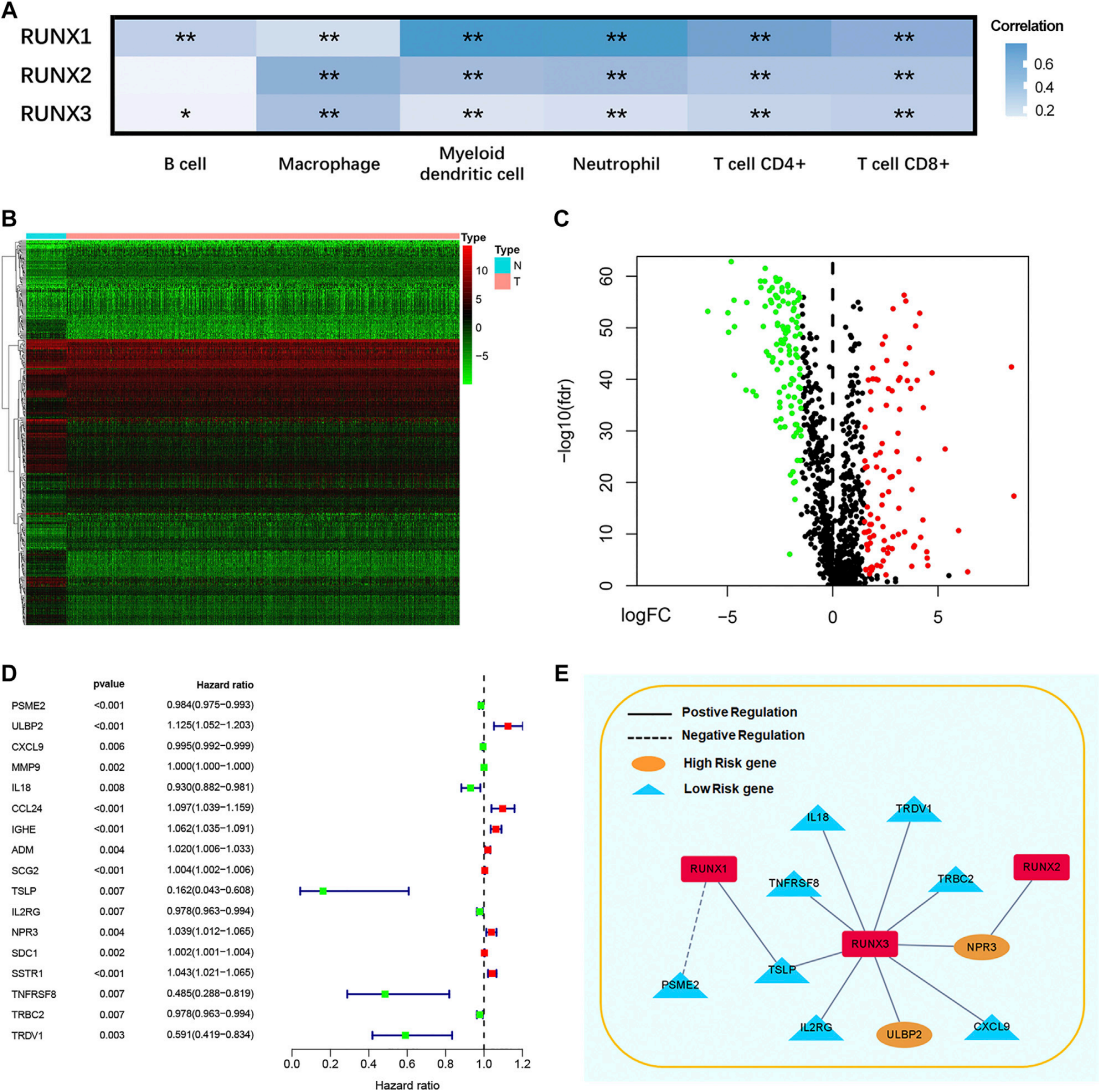
The correlation between different RUNXs and immune cell infiltration in breast cancer **(A)**. Heatmap **(B)** and volcanic maps **(C)** of 370 differentially expressed immune-related RUNXs genes from TCGA. The forest plot of hazard ratios demonstrated the prognostic values of immune-related genes (IRGs) **(D)**. The dash line was used to mark the location of HR = 1. The red box represented the adverse prognostic factor; Blue box represented the favorable prognostic factor. Regulation network of the RUNXs related to immune activities **(E)**. Genes showed positive correlation with RUNX were in solid line, and the genes showed negative correlation with the RUNX were in dotted line. High risk genes were in yellow, and low risk genes were in blue.

As shown in this study, we explored the correlation between RUNX family and immune cell infiltration by using the TIMER database. The expression of RUNX Family was in connection with the infiltration of B cells, macrophages, myeloid dendritic cells neutrophils, CD4^+^ T cells, and CD8^+^ T cells while RUNX2 was not connected with B cells (Figure 3A). We obtained 2,483 immunologically relevant genes from the ImmPort database considering that RUNX family was associated with immune infiltration. Then, we compared the expression of the difference between tumor and normal tissues *via* TCGA databases, and 370 differentially expressed immune-related genes were filtrated. The volcano map and heatmap were plotted to visualize these genes (Figures 3B,C). A forest map identified 17 genes connected with prognosis in breast cancer patients according to 2483 immunologically relevant genes (Figure 3D). Subsequently, the regulatory network of RUNX and immune genes were mapped to probe the association between RUNX family and these genes (Figure 3E). RUNX1 negatively regulated PSME2 and positively regulated TSLP, but they were both low-risk genes. RUNX2 positively regulated NPR3, a high-risk gene. Low-risk genes such as IL18, TNFRSF8, TSLP, IL2RG, TRDV1, TRBC2, and CXCL9 were positively regulated by RUNX3, and at the same time, RUNX3 negatively regulated high-risk genes like NPR3 and ULBP2.

### Construction of prognostic models

**FIGURE 4:**
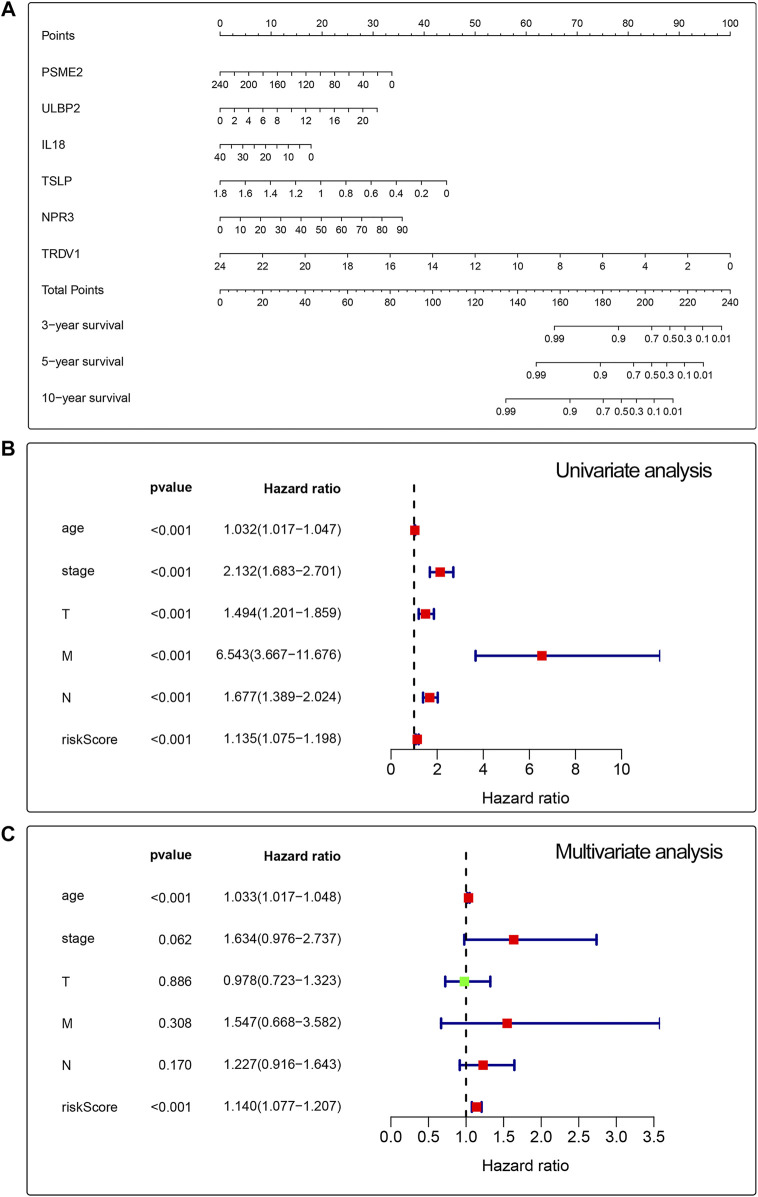
Univariate and multivariate analyses for breast cancer using the Cox regression model. A prognostic risk score model was established using independent risk factors **(A)**. Forest plots of univariate and multivariate Cox regression analyses had significant prognostic significance **(B,C)**.

We built a prognosis-related risk score model by using the independent risk factors to provide a clinically appropriate method for predicting the OS probability of patients with breast cancer. We screened out 10 genes that might be regulated by the RUNX family by analyzing the potential relationship between 17 prognosis-related genes and the RUNX family. Then, we performed cross-validation on the expression and prognosis of genes to prevent overfitting in the prognostic model. Based on the results, we obsoleted the genes with high correlation and obtained the most suitable number of genes with the smallest error to construct the prognostic model. Finally, we obtained the predictors including PSME2, ULBP2, IL18, TSLP, NPR3, and TRDV1 to build a prognosis-related risk score model (Figure 4A). Then we performed univariate Cox to analyze the relationship between risk value and clinicopathological parameters and overall survival (Figure 4B), T stage [HR = 1.494, 95%CI (1.201-1.859)], N stage [HR = 1.677, 95%CI (1.389-2.024)], M stage [HR = 6.543, 95%CI (3.667-11.676)] and risk value [HR = 1.135, 95%CI (1.075-1.198)] were related to prognosis. Multivariate Cox regression analysis further showed (Figure 4C) that T stage, N stage, M stage, and risk score can independently predict the prognosis of breast cancer patients.

### Validation of the prognostic model

**FIGURE 5:**
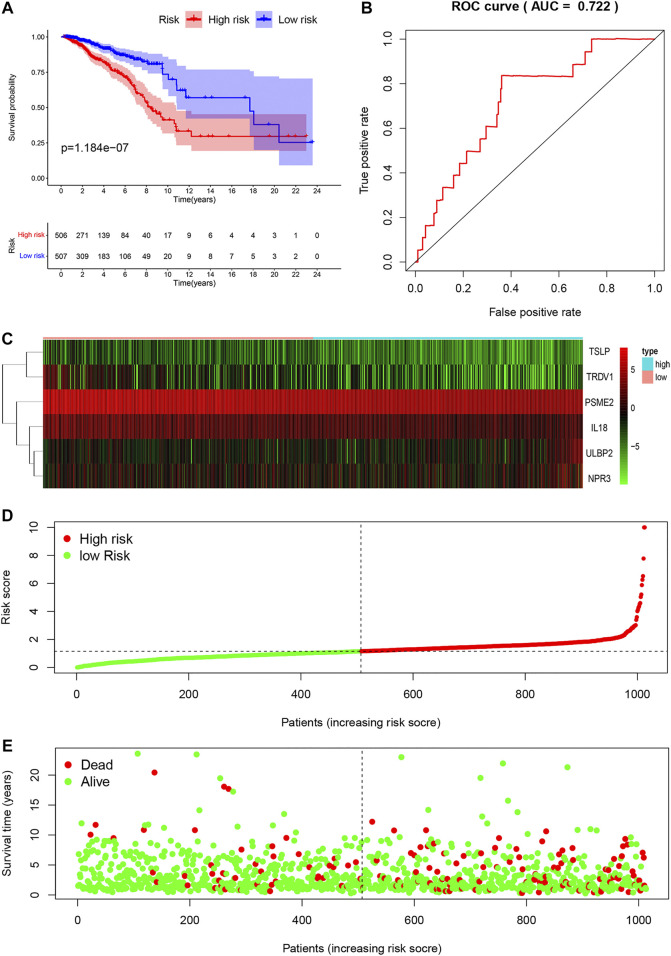
Characteristics of prognostic gene signatures. Survival analysis of the association between the independent risk factors and overall survival in breast cancer patients. Breast cancer patients were divided into high-risk (red line) and low-risk (blue line) groups according to their signature scores **(A)**. ROC analysis was performed to find out the most optimal cutoff value to divide the breast cancer patients into high risk and low risk group **(B)**. Heatmap of the expression profiles of RUNX family genes in different immune-genes, including TSLP, TRDV1, PSME2, IL18, ULBP2 and NPR3. Red represented high-expression, and blue represented low-expression **(C)**. The risk score distribution and the survival status of breast cancer patients **(D,E)**.

We divided 1013 breast cancer samples with clinical information from TCGA databases into the low-risk group (N = 507) and the high-risk group (N = 506) according to clinical risk factors. Generally, patients with higher risk scores had a bad prognosis than those with lower risk scores (Figure 5A). We figured out the value of AUC was 0.722 through the ROC analysis to assess the prognostic accuracy of the metabolic signature, indicating that this nomogram had high predictive accuracy (Figure 5B). The heatmap showing gene expression profiles in high-risk and low-risk groups were drawn to display the distribution of the gene expression differences (Figure 5C). The distribution of survival status showed that the survival time and the number of patients in the high-risk group were lower than those in the low-risk group (Figures 5D,E).

### Relationship between RUNX family and immune infiltrated

**FIGURE 6:**
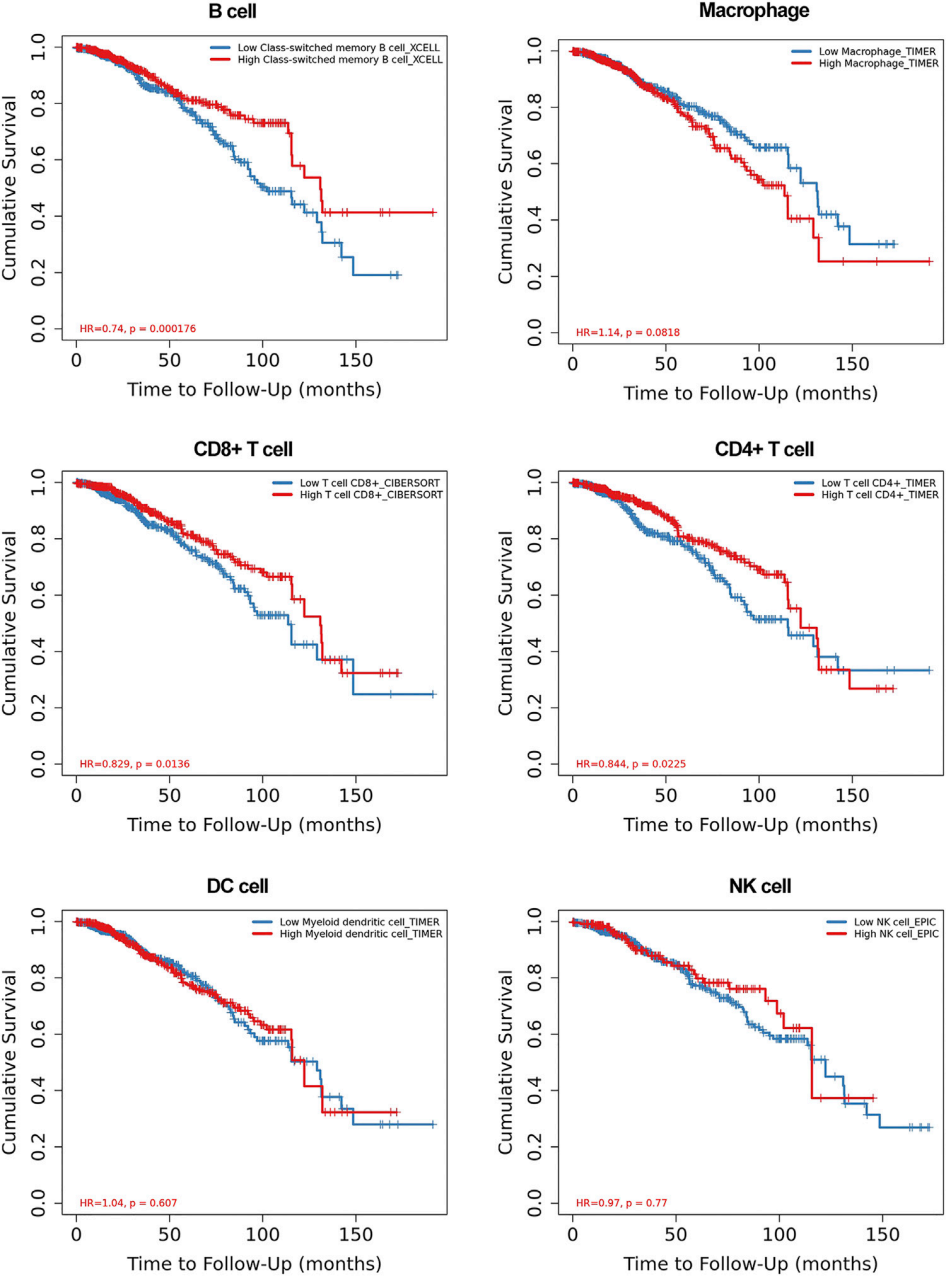
The relationship between the immune cell infiltration and clinical outcome of breast cancer patients.

To clarify whether the RUNX family is indeed associated with immune infiltration, we further explored the relationship between immune cell infiltration and the clinical outcome. The level of immune cells was closely related to the proliferation and development of cancer cells. In this study, the correlation between RUNX members and immune cell infiltration was explored by using the TIMER database. We discovered that the clinical outcomes (age, stage) of breast cancer patients were strongly correlated with immune cell infiltration. In particular, low immune infiltration of B cells and T cells often predicted a poor prognosis while macrophages have the opposite effect (Figure 6). Further, the expression of RUNX2 was associated with the infiltration of CD4 +T cells, neutrophils, B cells, CD8 + T cells, macrophages, and dendritic cells in breast cancer. RUNX1 was positively associated with the infiltration of CD4 +T cells, neutrophils, B cells, CD8 + T cells, and macrophages in breast cancer patients, but there was no significant correlation with dendritic cells. Concerning RUNX3, except for B cells, the remaining five host immune cells have a positive correlation with RUNX3 (Supplementary Figure S1). These results suggested that the RUNX family influences the prognosis of breast cancer by interacting with immune cells infiltration.

### RUNX3 could target and regulate ULBP2 and TRDV1

**FIGURE 7:**
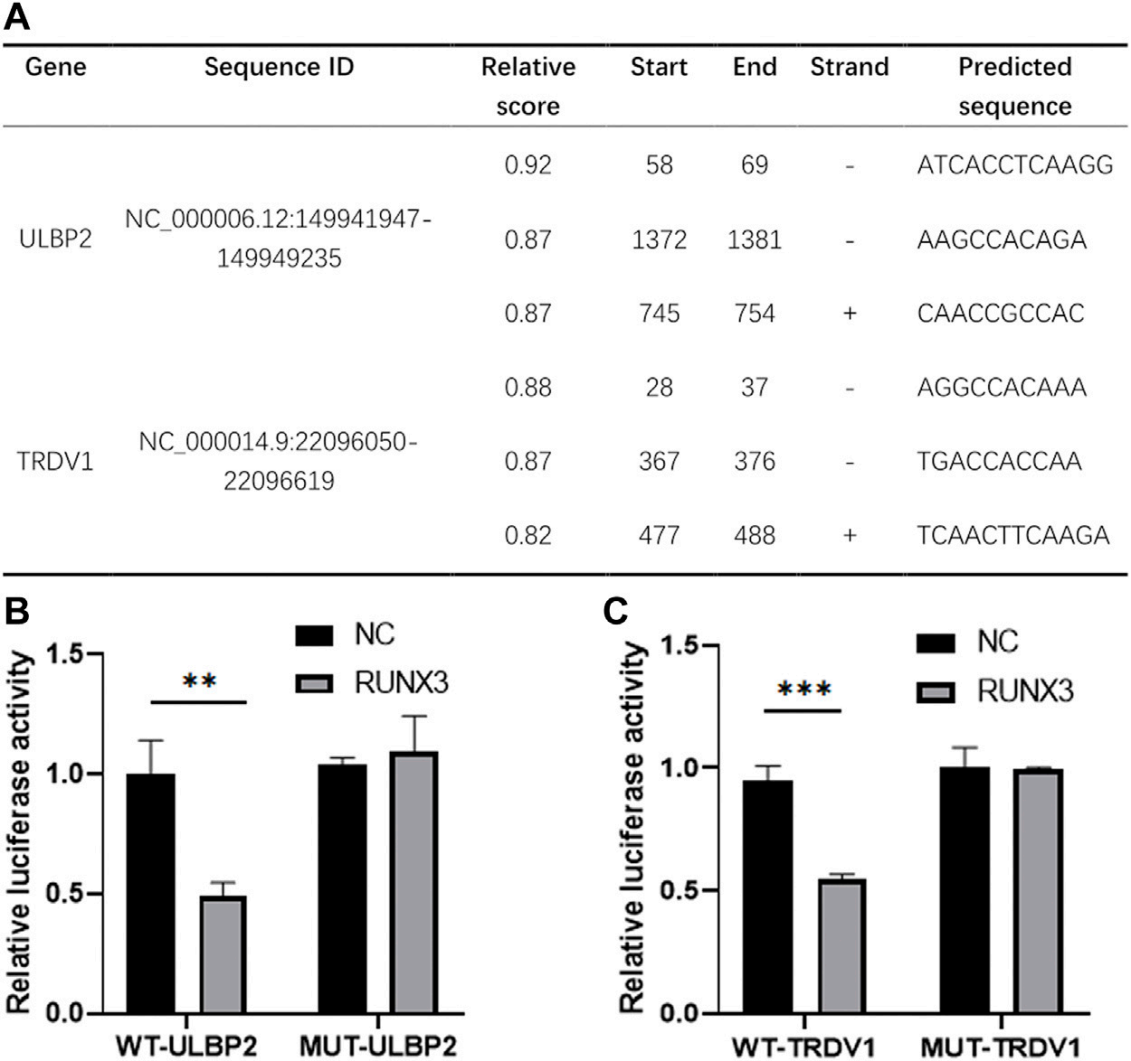
Transcription factor RUNX3 target regulated ULBP2 and TRDV1. JASPER website was used to predict the binding sites of RUNX3 in ULBP2 and TRDV1 promoter region **(A)**. Dual-Luciferase reporter assay was used to verify the binding sequence **(B)**.

In those risk predictors, the level of TSLP expression was correlated with breast cancer growth and metastasis (Kuan and Ziegler, 2018; Shi et al., 2020). PSME2, IL18 and NPR3 have been reported to have a close correlation with the proliferation, invasion, and migration of various tumors including breast cancer (Li et al., 2016a; Gu et al., 2018; Ramdas et al., 2019; Wang et al., 2021). However, the expression and function of ULBP2 and TRDV1 have not been reported. Based on the correlation analysis and research status, we chose RUNX3 and the high-risk gene ULBP2 and the low-risk gene TRDV1 as the research object. We used the JASPAR database to predict the possible binding sites of the transcription factor RUNX3 in the promoter region of ULBP2 and TRDV (Figure 7A). By selecting the sequence of potential loci with the highest score, we designed the dual-luciferase reporter assay to verify the relationship between RUNX3 and the immune genes. The results showed that ULBP2 and TRDV1 were target-regulated by RUNX3 (Figure 7B).

### Function analysis of ULBP2 and TRDV1 in breast cancer cells

Based on the above results, to explore the relationship between RUNX family and immune genes, we made further research on the immune genes ULBP2 and TRDV1. We applied CCK-8, Edu, Colony formation and Wound healing assays to detect the functions of ULBP2 and TRDV1 in breast cancer cells.

**FIGURE 8:**
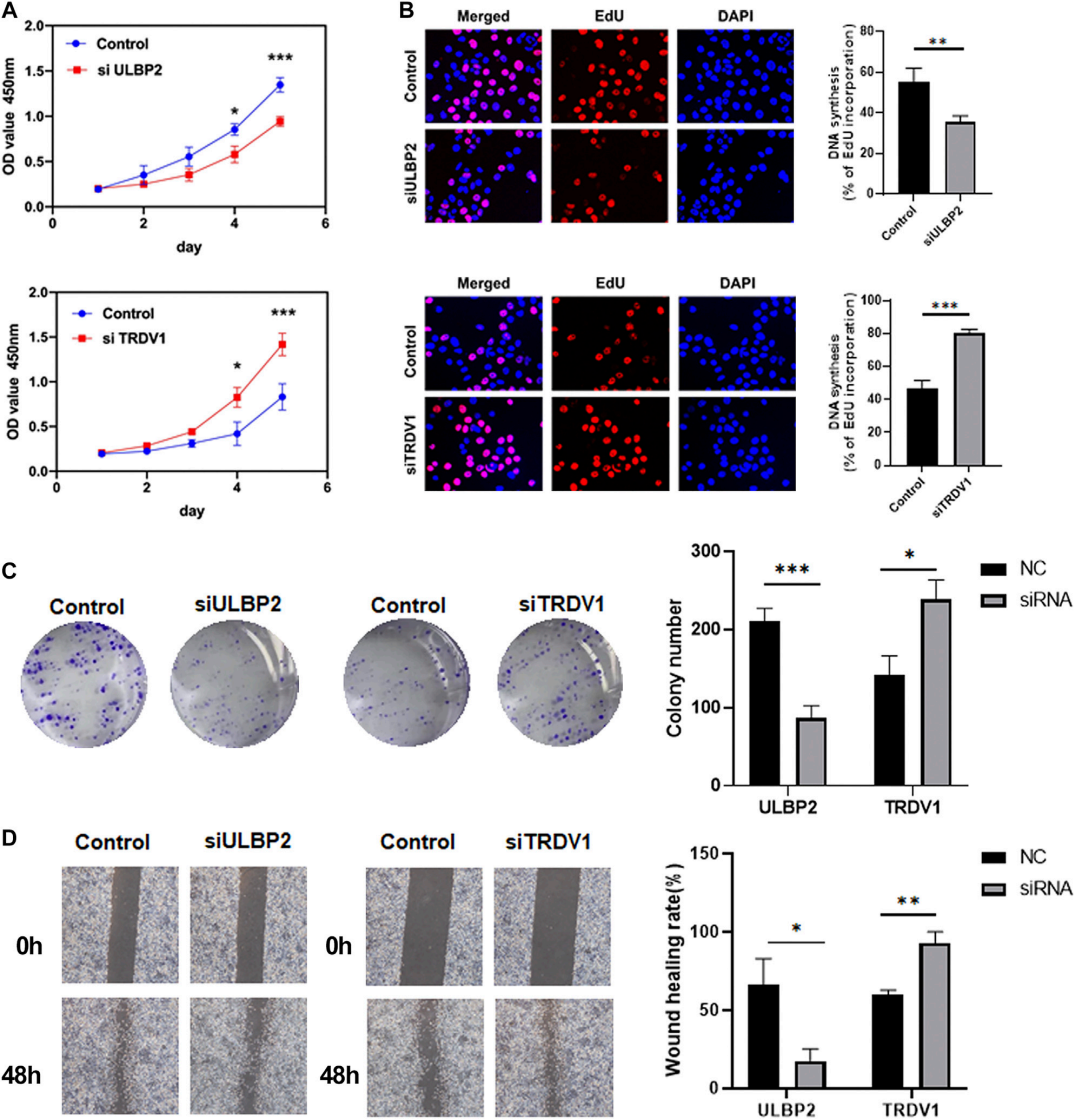
ULBP2 knockdown inhibited cell proliferation, migration and invasion in vitro, TRDV1 knockdown have the opposite functions. CCK-8 assay was performed to determine the proliferation of MDA-MB-231 cells infected with ULBP2 and TRDV1 lentivirus **(A)**. Edu assays were used to evaluate the effect of ULBP2 and TRDV1 knockdown on breast cancer cell proliferation **(B)**. Colony formation assays were performed to evaluate the proliferation of cells with ULBP2 and TRDV1 knockdown **(C)**. The effect of ULBP2 and TRDV1 knockdown on cell migration was examined by wound healing assay **(D)**.

We assessed the impact of ULBP2 and TRDV1 knockdown on breast cancer cell functions. The CCK-8 and colony formation assays revealed that down-regulation of ULBP2 significantly suppressed cell proliferation of MDA-MB-231. However, the TRDV1 knockdown promoted the proliferation of cells (Figures 8A,C). Edu assays showed that the low expression of ULBP2 formed less DNA replication level than the control group (Figure 8B). Moreover, different scratch healing rates in wound healing analysis showed that motile ability was significantly enhanced after the knockdown of TRDV1 and decreased after knocking down ULBP2 (Figure 8D).

## Discussion

In recent years, the role RUNX family in various tumors has attracted extensive attention, and relevant studies have confirmed their functions. Although the role of RUNX family has been partially confirmed in the tumorigenesis and prognosis of several cancers (Samarakkody et al., 2020; Hass et al., 2021), the distinct roles of RUNX family members in breast cancer remain to be elucidated. More and more studies have shown that there are many kinds of immune cell infiltrates in breast cancer tissues, these immune cells affect the pathogenesis and metastasis of breast cancer through a variety of signaling pathways, which may affect the prognosis of patients. Hence, a further bioinformatics analysis of breast cancer was performed to expound on the functions of RUNX family, especially its relationship with immune regulation. Currently, the relationship between the immune system of RUNX family members in breast cancer has not been fully studied, so our study comprehensively investigated the mRNA expression, mutation, and prognostic values (OS and RFS) of different RUNXs factors in breast cancer and especially for the first time to research their relationship with immune cell infiltration. Our findings could enhance the accuracy of predicting prognosis for patients with breast cancer.

In the progression of cancers, RUNX family has been involved in the regulation of different carcinogenic processes and signaling pathways with cancer (Sweeney et al., 2020). Hong et al. founded that RUNX1 reduces breast cancer cell migration and invasion *in vitro* and tumor growth *in vivo*, and also founded that high levels of RUNX1 expression can suppress metastasis, treatment resistance, and tumor recurrence in breast cancer (Hong et al., 2018). Co-expression of RUNX1 or RUNX3 significantly suppressed modulate YAP-mediated oncogenic phenotypes, and inhibited breast cancer progression (Niu et al., 2012). RUNX2 was overexpressed in breast and prostate cancer and associated with increased metastatic capacity. In contrary to RUNX1 and RUNX3, RUNX2 promoted cancer cell recruitment and adhesion in bone and further facilitated bone colonization (Li et al., 2016b). Meanwhile, RUNX2 expression was increased by increasing the expression of vascular endothelial growth factor, matrix metalloproteases (MMP2, MMP9, and MMP13), and bone sialoprotein (BSP) in breast cancer metastatic cells (Dowdy et al., 2010). Each RUNX factor has been researched in isolation without reference to the other genes in the family because of their lineage-specific expression. However, there was an increasing realization that RUNX genes’ functions are complementary and must be studied at the same time (Ito and Miyazono, 2003; Ito et al., 2015). Experimental evidence has revealed that RUNX genes have exhibited oncogenes or tumor suppressor genes (Kilbey et al., 2008; Blyth et al., 2010).

Tumor-infiltrating immune cells (TIICs) in the tumor microenvironment (TME) are becoming an increasingly important role in affecting tumorigenesis, progression and metastasis, and immune cells level is closely associated with the proliferation and progression of the cancer cell. Studies had pointed out that RUNX family proteins are significantly involved in the development, organization, and function of the mammalian immune system (Zusso et al., 2012). Macrophages and monocytes (Edin et al., 2012; Tong et al., 2021) had been demonstrated to be related to poor prognosis while T cells (Sato et al., 2005) meant better prognosis in various cancers. RUNX family also took part in the activation of peripheral T and B cells within these tissues through mediating DC maturation for antigen presentation (Fainaru et al., 2004; Fainaru et al., 2005; Satpathy et al., 2014). Therefore, in this study, we explored the association between the expression of RUNX family and immune cell infiltration. This study founded that the expression of RUNX2 was positively related to the immune infiltration of CD4^+^ T cells, CD8^+^ T cells, B cells, neutrophils, DC cells, and macrophages in the breast cancer microenvironment. Meanwhile, we discovered that the expression of RUNX1 was significantly correlated with the biomarkers of CD4^+^ T cells, CD8^+^ T cells, B cells, neutrophils, and macrophages. In addition, RUNX3 was not significantly correlated with B cells, but also positively correlated with other immune cells. As described in our study, the risk model related to RUNX proteins was negatively correlated with immune infiltration, indicating that the RUNX family exerted a pro-cancer effect in breast cancer. In previous studies, the mRNA and protein expression of RUNX family has been confirmed to be higher in breast cancer tissues than that in adjacent tissues (Gao and Zhou, 2021), and its expression was markedly correlated with clinical prognosis in breast cancer patients. Then, both univariate and multivariate logistic regression analyses demonstrated that 6 genes regulated by the RUNX family had a significant relationship with the prognosis of breast cancer patients, including PSME2, ULBP2, IL18, TSLP, NPR3, and TRDV1. Based on these results, we used the JASPAR database to explore the relationship between RUNX3 and ULBP2 and TRDV1, the results showed that the RUNX3 target regulated the two immune genes. In addition, we made further research on the functions of immune genes ULBP2 and TRDV1, and the results showed that down-regulation of ULBP2 suppressed cell proliferation and TRDV1 has the opposite functions.

Subsequently, we used the prognostic model constructed by these genes to divide breast cancer patients into subgroups for comparative analysis, further verifying the independent predictive value in breast cancer patients. As a result, a statistically significant difference in the overall survival rate was found between the two subtypes.

In conclusion, we developed and validated a prognostic model for patients with breast cancer. The results showed that our model could promote the development of the prognostic assessment, improve the accuracy of predicting the prognosis of breast cancer patients, and is expected to become a new independent biomarker for the prognosis of breast cancer patients. In this study, though, we discussed the important role of RUNX family members in breast cancer, however, the limitations of this study should be noted. First, we analyzed and evaluated our gene family by using limited data and clinical information from the genomic commons data. In addition, it is critical to verify the functional characteristics and molecular mechanisms of RUNX family through biological experiments and clinical studies.

## Data Availability

The original contributions presented in the study are included in the article/Supplementary Materials, further inquiries can be directed to the corresponding authors.

## References

[B1] Ashe H. Krakowiak P. Hasterok S. Sleppy R. Roller D. G. Gioeli D. (2021). Role of the runt-related transcription factor (RUNX) family in prostate cancer. FEBS J. 288 (21), 6112–6126. 10.1111/febs.15804 33682350

[B2] Blyth K. Vaillant F. Jenkins A. McDonald L. Pringle M. A. Huser C. (2010). Runx2 in normal tissues and cancer cells: A developing story. Blood Cells Mol. Dis. 45 (2), 117–123. 10.1016/j.bcmd.2010.05.007 20580290

[B3] Bowcock A. (2005). The genetics of psoriasis and autoimmunity. Annu. Rev. Genomics Hum. Genet. 6, 93–122. 10.1146/annurev.genom.6.080604.162324 16124855

[B4] Dowdy C. Xie R. Frederick D. Hussain S. Zaidi S. K. Vradii D. (2010). Definitive hematopoiesis requires Runx1 C-terminal-mediated subnuclear targeting and transactivation. Hum. Mol. Genet. 19 (6), 1048–1057. 10.1093/hmg/ddp568 20035012 PMC2830828

[B5] Edin S. Wikberg M. L. Dahlin A. M. Rutegard J. Oberg A. Oldenborg P. A. (2012). The distribution of macrophages with a M1 or M2 phenotype in relation to prognosis and the molecular characteristics of colorectal cancer. PloS one 7 (10), e47045. 10.1371/journal.pone.0047045 23077543 PMC3471949

[B6] Estecha A. Aguilera-Montilla N. Sanchez-Mateos P. Puig-Kroger A. (2012). RUNX3 regulates intercellular adhesion molecule 3 (ICAM-3) expression during macrophage differentiation and monocyte extravasation. PloS one 7 (3), e33313. 10.1371/journal.pone.0033313 22479382 PMC3315569

[B7] Fainaru O. Shseyov D. Hantisteanu S. Groner Y. (2005). Accelerated chemokine receptor 7-mediated dendritic cell migration in Runx3 knockout mice and the spontaneous development of asthma-like disease. Proc. Natl. Acad. Sci. U. S. A. 102 (30), 10598–10603. 10.1073/pnas.0504787102 16027362 PMC1180803

[B8] Fainaru O. Woolf E. Lotem J. Yarmus M. Brenner O. Goldenberg D. (2004). Runx3 regulates mouse TGF-beta-mediated dendritic cell function and its absence results in airway inflammation. EMBO J. 23 (4), 969–979. 10.1038/sj.emboj.7600085 14765120 PMC380997

[B9] Fritz A. J. Hong D. Boyd J. Kost J. Finstaad K. H. Fitzgerald M. P. (2020). RUNX1 and RUNX2 transcription factors function in opposing roles to regulate breast cancer stem cells. J. Cell. Physiol. 235 (10), 7261–7272. 10.1002/jcp.29625 32180230 PMC7415511

[B10] Gao L. Zhou F. (2021). Comprehensive analysis of RUNX and TGF-β mediated regulation of immune cell infiltration in breast cancer. Front. Cell Dev. Biol. 9, 730380. 10.3389/fcell.2021.730380 34485309 PMC8416425

[B11] Gu L. Lu L. Zhou D. Liu Z. (2018). Long noncoding RNA BCYRN1 promotes the proliferation of colorectal cancer cells *via* up-regulating NPR3 expression. Cell. Physiol. biochem. 48 (6), 2337–2349. 10.1159/000492649 30114690

[B12] Hass M. Brissette D. Parameswaran S. Pujato M. Donmez O. Kottyan L. C. (2021). Runx1 shapes the chromatin landscape *via* a cascade of direct and indirect targets. PLoS Genet. 17 (6), e1009574. 10.1371/journal.pgen.1009574 34111109 PMC8219162

[B13] Hong D. Fritz A. J. Finstad K. H. Fitzgerald M. P. Weinheimer A. Viens A. L. (2018). Suppression of breast cancer stem cells and tumor growth by the RUNX1 transcription factor. Mol. Cancer Res. 16 (12), 1952–1964. 10.1158/1541-7786.MCR-18-0135 30082484 PMC6289193

[B14] Huang B. Qu Z. Ong C. W. Tsang Y. H. N. Xiao G. Shapiro D. (2012). RUNX3 acts as a tumor suppressor in breast cancer by targeting estrogen receptor α. Oncogene 31 (4), 527–534. 10.1038/onc.2011.252 21706051 PMC3697905

[B15] Huang S. Li D. Zhuang L. Sun L. Wu J. (2021). Identification of arp2/3 complex subunits as prognostic biomarkers for hepatocellular carcinoma. Front. Mol. Biosci. 8, 690151. 10.3389/fmolb.2021.690151 34307456 PMC8299467

[B16] Ito Y. Bae S. C. Chuang L. S. (2015). The RUNX family: developmental regulators in cancer. Nat. Rev. Cancer 15 (2), 81–95. 10.1038/nrc3877 25592647

[B17] Ito Y. Miyazono K. (2003). RUNX transcription factors as key targets of TGF-beta superfamily signaling. Curr. Opin. Genet. Dev. 13 (1), 43–47. 10.1016/s0959-437x(03)00007-8 12573434

[B18] Kilbey A. Terry A. Cameron E. R. Neil J. C. (2008). Oncogene-induced senescence: an essential role for runx. Cell cycleGeorget. Tex.) 7 (15), 2333–2340. 10.4161/cc.6368 PMC2562501 18677118

[B19] Komori T. (2020). Molecular mechanism of runx2-dependent bone development. Mol. Cells 43 (2), 168–175. 10.14348/molcells.2019.0244 31896233 PMC7057844

[B20] Kuan E. L. Ziegler S. F. (2018). A tumor-myeloid cell axis, mediated *via* the cytokines IL-1α and TSLP, promotes the progression of breast cancer. Nat. Immunol. 19 (4), 366–374. 10.1038/s41590-018-0066-6 29556001 PMC5864553

[B21] Li K. Wei L. Huang Y. Wu Y. Su M. Pang X. (2016). Leptin promotes breast cancer cell migration and invasion *via* IL-18 expression and secretion. Int. J. Oncol. 48 (6), 2479–2487. 10.3892/ijo.2016.3483 27082857

[B22] Li X. Q. Lu J. T. Tan C. C. Wang Q. S. Feng Y. M. (2016). RUNX2 promotes breast cancer bone metastasis by increasing integrin α5-mediated colonization. Cancer Lett. 380 (1), 78–86. 10.1016/j.canlet.2016.06.007 27317874

[B23] Liu H. Chen C. Ma D. Li Y. Yin Q. Li Q. (2020). Inhibition of PIM1 attenuates the stem cell-like traits of breast cancer cells by promoting RUNX3 nuclear retention. J. Cell. Mol. Med. 24 (11), 6308–6323. 10.1111/jcmm.15272 32307917 PMC7294145

[B24] Liu J. Li C. Jiang Y. Wan Y. Zhou S. Cheng W. (2018). Tumor-suppressor role of miR-139-5p in endometrial cancer. Cancer Cell Int. 18, 51. 10.1186/s12935-018-0545-8 29618950 PMC5879796

[B25] Mevel R. Draper J. E. Lie-A-Ling M. Kouskoff V. Lacaud G. (2019). RUNX transcription factors: Orchestrators of development. Development 146 (17), 148296. 10.1242/dev.148296 31488508

[B26] Ni M. Zhao Y. Zhang W. J. Jiang Y. J. Fu H. Huang F. (2020). microRNA-802 accelerates hepatocellular carcinoma growth by targeting RUNX3. J. Cell. Physiol. 235 (10), 7128–7135. 10.1002/jcp.29611 32003017

[B27] Niu D. F. Kondo T. Nakazawa T. Oishi N. Kawasaki T. Mochizuki K. (2012). Transcription factor Runx2 is a regulator of epithelial-mesenchymal transition and invasion in thyroid carcinomas. Lab. Invest. 92 (8), 1181–1190. 10.1038/labinvest.2012.84 22641097

[B28] Otalora-Otalora B. A. Henriquez B. Lopez-Kleine L. Rojas A. (2019). RUNX family: Oncogenes or tumor suppressors (Review). Oncol. Rep. 42 (1), 3–19. 10.3892/or.2019.7149 31059069 PMC6549079

[B29] Puig-Kröger A. Sanchez-Elsner T. Ruiz N. Andreu E. J. Prosper F. Jensen U. B. (2003). RUNX/AML and C/EBP factors regulate CD11a integrin expression in myeloid cells through overlapping regulatory elements. Blood 102 (9), 3252–3261. 10.1182/blood-2003-02-0618 12855590

[B30] Qin X. Jiang Q. Nagano K. Moriishi T. Miyazaki T. Komori H. (2020). Runx2 is essential for the transdifferentiation of chondrocytes into osteoblasts. PLoS Genet. 16 (11), e1009169. 10.1371/journal.pgen.1009169 33253203 PMC7728394

[B31] Ramdas P. Radhakrishnan A. K. Abdu Sani A. A. Kumari M. Anandha Rao J. S. Abdul-Rahman P. S. (2019). Advancing the role of gamma-tocotrienol as proteasomes inhibitor: A quantitative proteomic analysis of MDA-MB-231 human breast cancer cells. Biomolecules 10 (1), 19. 10.3390/biom10010019 PMC7022772 31877708

[B32] Rooney N. Riggio A. I. Mendoza-Villanueva D. Shore P. Cameron E. R. Blyth K. (2017). Runx genes in breast cancer and the mammary lineage. Adv. Exp. Med. Biol. 962, 353–368. 10.1007/978-981-10-3233-2_22 28299668

[B33] Samarakkody A. Shin N. Cantor A. (2020). Role of RUNX family transcription factors in DNA damage response. Mol. Cells 43 (2), 99–106. 10.14348/molcells.2019.0304 32024352 PMC7057837

[B34] Sato E. Olson S. H. Ahn J. Bundy B. Nishikawa H. Qian F. (2005). Intraepithelial CD8+ tumor-infiltrating lymphocytes and a high CD8+/regulatory T cell ratio are associated with favorable prognosis in ovarian cancer. Proc. Natl. Acad. Sci. U. S. A. 102 (51), 18538–18543. 10.1073/pnas.0509182102 16344461 PMC1311741

[B35] Satpathy A. Briseno C. G. Cai X. Michael D. G. Chou C. Hsiung S. (2014). Runx1 and Cbfβ regulate the development of Flt3+ dendritic cell progenitors and restrict myeloproliferative disorder. Blood 123 (19), 2968–2977. 10.1182/blood-2013-11-539643 24677539 PMC4014839

[B36] Seo W. Taniuchi I. (2020). The roles of RUNX family proteins in development of immune cells. Mol. Cells 43 (2), 107–113. 10.14348/molcells.2019.0291 31926543 PMC7057832

[B37] Shi H. Zhao L. Guo X. Fang R. Zhang H. Dong G. (2020). Arctigenin attenuates breast cancer progression through decreasing GM-CSF/TSLP/STAT3/β-Catenin signaling. Int. J. Mol. Sci. 21 (17), 6357. 10.3390/ijms21176357 PMC7503539 32887217

[B38] Song J. Liu Y. Wang T. Li B. Zhang S. (2020). MiR-17-5p promotes cellular proliferation and invasiveness by targeting RUNX3 in gastric cancer. Biomed. Pharmacother. 128, 110246. 10.1016/j.biopha.2020.110246 32447210

[B39] Sood R. Kamikubo Y. Liu P. (2017). Role of RUNX1 in hematological malignancies. Blood 129 (15), 2070–2082. 10.1182/blood-2016-10-687830 28179279 PMC5391618

[B40] Sun C. C. Li S. J. Hu W. Zhang J. Zhou Q. Liu C. (2019). Retracted: Comprehensive analysis of the expression and prognosis for E2Fs in human breast cancer. Mol. Ther. 27 (6), 1153–1165. 10.1016/j.ymthe.2019.03.019 31010740 PMC6554685

[B41] Sung H. Ferlay J. Siegel R. L. Laversanne M. Soerjomataram I. Jemal A. (2021). Global cancer statistics 2020: GLOBOCAN estimates of incidence and mortality worldwide for 36 cancers in 185 countries. Ca. Cancer J. Clin. 71 (3), 209–249. 10.3322/caac.21660 33538338

[B42] Sweeney K. Cameron E. R. Blyth K. (2020). Complex interplay between the RUNX transcription factors and wnt/β-catenin pathway in cancer: A tango in the night. Mol. Cells 43 (2), 188–197. 10.14348/molcells.2019.0310 32041394 PMC7057843

[B43] Tang Z. Li C. Kang B. Gao G. Li C. Zhang Z. (2017). Gepia: a web server for cancer and normal gene expression profiling and interactive analyses. Nucleic Acids Res. 45, W98–W102. 10.1093/nar/gkx247 28407145 PMC5570223

[B44] Tong N. He Z. Ma Y. Wang Z. Huang Z. Cao H. (2021). Tumor associated macrophages, as the dominant immune cells, are an indispensable target for immunologically cold tumor-glioma therapy? Front. Cell Dev. Biol. 9, 706286. 10.3389/fcell.2021.706286 34368156 PMC8337013

[B45] Uhlén M. Fagerberg L. Hallstrom B. M. Lindskog C. Oksvold P. Mardinoglu A. (2015). Proteomics. Tissue-based map of the human proteome. Sci. (New York, N.Y.) 347 (6220), 1260419. 10.1126/science.1260419 25613900

[B46] Vishal M. Swetha R. Thejaswini G. Arumugam B. Selvamurugan N. (2017). Role of Runx2 in breast cancer-mediated bone metastasis. Int. J. Biol. Macromol. 99, 608–614. 10.1016/j.ijbiomac.2017.03.021 28268169

[B47] Voon D. Hor Y. Ito Y. (2015). The RUNX complex: reaching beyond haematopoiesis into immunity. Immunology 146 (4), 523–536. 10.1111/imm.12535 26399680 PMC4693896

[B48] Wang X. Wu F. Deng Y. Chai J. Zhang Y. He G. (2021). Increased expression of PSME2 is associated with clear cell renal cell carcinoma invasion by regulating BNIP3mediated autophagy. Int. J. Oncol. 59 (6), 106. 10.3892/ijo.2021.5286 34779489 PMC8651225

[B49] Zusso M. Methot L. Lo R. Greenhalgh A. D. David S. Stifani S. (2012). Regulation of postnatal forebrain amoeboid microglial cell proliferation and development by the transcription factor Runx1. J. Neurosci. 32 (33), 11285–11298. 10.1523/JNEUROSCI.6182-11.2012 22895712 PMC6621177

