# Supplementary material for: RUNX regulated immune-associated genes predicts prognosis in breast cancer
Source: Front Genet. 2022 Aug 26;13:960489. doi: 10.3389/fgene.2022.960489 (PMC9459239; doi:10.3389/fgene.2022.960489)
Supplement: Supplementary file 2 [file Table1.DOC]

https://www.jianguoyun.com/p/DcUrUEgQk4LUChju08MEIAA
